# Longitudinal study of meningococcal carriage rates in university entrants living in a dormitory in South Korea

**DOI:** 10.1371/journal.pone.0244716

**Published:** 2021-01-28

**Authors:** Heun Choi, Hyuk Min Lee, Woonji Lee, Jun Hyoung Kim, Hye Seong, Jung Ho Kim, Jin Young Ahn, Su Jin Jeong, Nam Su Ku, Joon-Sup Yeom, Kyungwon Lee, Hee Soo Kim, Philipp Oster, Jun Yong Choi

**Affiliations:** 1 Department of Internal Medicine, National Health Insurance Service Ilsan Hospital, Goyang, Republic of Korea; 2 Department of Laboratory Medicine, Yonsei University College of Medicine, Seoul, Republic of Korea; 3 Department of Internal Medicine and AIDS Research Institute, Yonsei University College of Medicine, Seoul, Republic of Korea; 4 Sanofi Pasteur, Seoul, Republic of Korea; 5 Sanofi Pasteur, Lyon, France; Carnegie Mellon University, UNITED STATES

## Abstract

University students, especially those living in dormitories, are known to have a high risk of invasive meningococcal disease. We performed a longitudinal study to investigate the change in *Neisseria meningitidis* carriage rates and identify the risk factors for carriage acquisition in university students in South Korea. We recruited university entrants who were admitted to a student dormitory. Pharyngeal swabs were taken from participants at baseline, 1 month, and 3 months, and the subjects completed a questionnaire. Culture and real-time polymerase chain reaction (PCR) for species-specific *ctrA* and *sodC* genes were performed. The cultured isolates or PCR-positive samples were further evaluated for epidemiologic characterization using serogrouping, PorA typing, FetA typing, and multilocus sequence typing (MLST). At the first visit, we enrolled 332 participants who were predominantly male (64.2%) with a median age of 19 years. Meningococcal carriage rates increased from 2.7% (95% confidence interval [CI] 0.9–4.4%) at baseline to 6.3% (95% CI 3.4–9.0%) at 1 month and 11.8% (95% CI 7.8–15.6%) at 3 months. Nongroupable isolates accounted for 50.0% of all isolates, with serogroup B being the next most prevalent (24.1%). In the study population, male sex (OR 2.613, 95% CI 1.145–5.961, *p* = 0.022) and frequent pub or club visits (OR 3.701, 95% CI 1.536–8.919, *p* = 0.004) were significantly associated with meningococcal carriage. Based on serotype and MLST analyses, six carriers transmitted meningococci to other study participants. *N*. *meningitidis* carriage rates among new university entrants who lived in a dormitory significantly increased within the first 3 months of dormitory stay, probably owing to the transmission of identical genotype among students. Based on the risk of meningococcal disease, meningococcal vaccination should be considered for students before dormitory admission.

## Introduction

Despite appropriate treatment, *Neisseria meningitidis* can cause life-threatening invasive meningococcal disease (IMD), including meningitis and/or sepsis [1]. The human pharynx is a unique reservoir of *N*. *meningitidis*, and meningococci spread between close contacts via aerosol droplets released during coughing [2]. *N*. *meningitidis* infection often results in asymptomatic colonization of the human nasopharynx. Moreover, meningococcal transmission is easier through close contact with a *N*. *meningitidis* carrier than by close contact with an infected individual with IMD, underlining the importance of asymptomatic carriers [3, 4].

While the overall carriage prevalence is estimated at 10%, the incidence rate of IMD varies between 0.5/100,000 in North America and up to 1,000/100,000 in epidemic settings [5, 6]. In industrialized countries, carriage rates increase gradually through childhood (4.5%), peak during adolescence and young adulthood (23.7%), and stabilize during adulthood (7.8%). In developing countries, however, carriage rates generally peak in early childhood [7]. The carriage rate is also influenced by social risk factors, including going to clubs, kissing, smoking, and living in closed or semi-closed environments such as university dormitories [8]. In South Korea, only limited data are available. Moreover, the IMD annual incidence is low (0.01–0.08/100,000) and it occurs sporadically without clusters, other than the outbreak of army basic training center [1, 9]. Although the Korea Centers for Disease Control and Prevention (KCDC) guidelines recommend vaccination for new dormitory entrants, little is known about vaccination and no educational institution requires it [10]. Given that few studies have analyzed the meningococcal carriage rates in South Korean young adults, especially students residing in dormitories, it is necessary to conduct an appropriate epidemiologic analysis. Considering the high mortality rate of the IMD, the recent increase in antibiotic resistance is also a problem [9, 11].

This study aimed to assess the sequential changes in *N*. *meningitidis* carriage rates over a 3-month period in students newly admitted to a university dormitory in South Korea. In addition, we investigated the dominant circulating serogroups, antimicrobial susceptibility, and factors related to meningococcal pharyngeal carriage.

## Methods

### Study population

This prospective observational study was conducted during the first semester (March–June 2018) of the year at a university dormitory in Incheon, South Korea. We included first-year students, who resided in the same dormitory and provided their informed consent. An advertising email was sent to all students, and desks were placed at the entrance of the dormitory with a consent form to recruit the applicants. We excluded students who had a history of meningococcal vaccination, completed military service (quadrivalent meningococcal conjugate vaccine [MCV4] vaccination has been mandatory in the South Korean army since 2012), had taken antibiotics 2 weeks prior to the study, had taken immunosuppressants or steroid drugs 2 weeks prior to the study, or had existing immunological or hematological disorder. These exclusion criteria did not apply to re-visiting applicants.

We obtained approval from the Institutional Review Board of Severance Hospital (4-2017-1095), and collected written informed consent from all participants.

### Collected data and specimens

Three visits were programmed for each participant; on dormitory admission, at 1 month, and at 3 months (end of the first semester). Participants completed the questionnaires during each visit, wherein the following data were collected: demographic characteristics; medical history including current medications; recent symptoms of upper respiratory tract infection (within the previous week); recent record of traveling abroad (within past 4 weeks); the number of club/pub visits (within past 2 weeks); smoking (active or not); alcohol consumption (frequency); sharing of cups and cigarettes (past 1 week); and the number of kisses (within past 4 weeks). Two oropharyngeal swabs, from the tonsils/the tonsillar fossa and the posterior pharynx, were collected simultaneously at each visit using BD CultureSwab MaxV Liquid Amies (Becton-Dickinson, USA) for conventional culture and molecular screening tests.

### Microbiological tests

**Fig 1:**
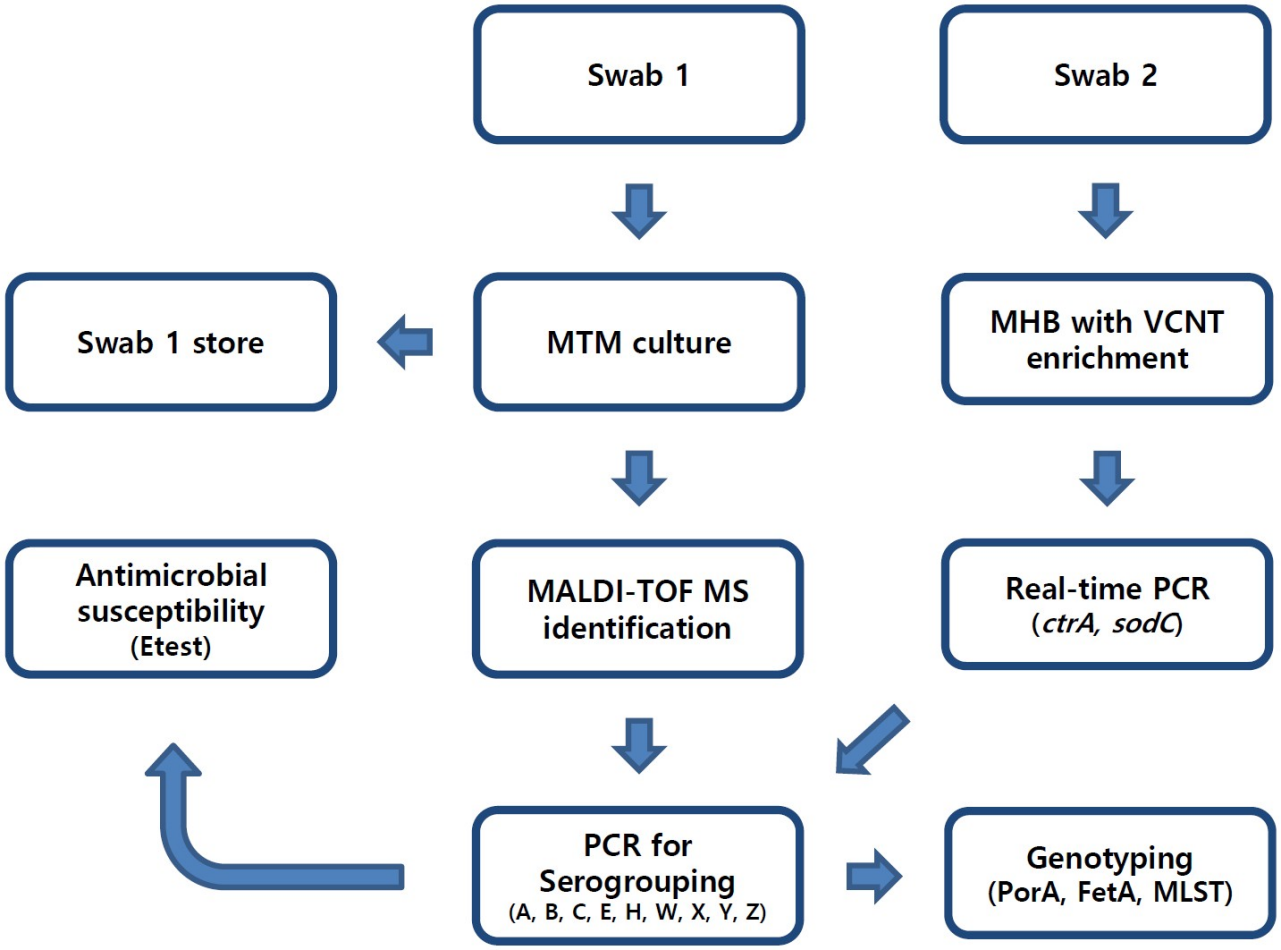
Schematic diagram for specimen processing. MHB, Mueller-Hinton broth; MLST, multilocus sequence typing; MTM, modified Thayer-Martin media; PCR, polymerase chain reaction; VCNT, vancomycin, colistin, nystatin, trimethoprim; MALDI-TOF MS, matrix-assisted laser desorption/ionisation-time of flight mass spectrometry.

One swab was inoculated into modified Thayer-Martin media and stored for future analysis. The second swab was inoculated into Mueller-Hinton broth containing vancomycin (3 mg/L), colistin (7.5 mg/L), nystatin (1,250 U/L), and trimethoprim (5 mg/L) for enrichment. The broth was then incubated overnight, part of it inoculated into modified Thayer-Martin media, and the rest stored for molecular screening. The inoculated Thayer-Martin media from the direct swab and enriched broth were both incubated for 72 hours to allow for *N*. *meningitidis* growth. Suspected colonies were identified at 24, 48, and 72 hours with MALDI-TOF mass spectrophotometry (Bruker Daltonics). All isolates were stored in a deep freezer at -70°C for further analysis. The stored enriched broth was tested for *N*. *meningitidis*-specific *ctrA* and *sodC* genes using real-time polymerase chain reaction (PCR) as previously described [12, 13]. We undertook epidemiologic characterization of *N*. *meningitidis* isolates or PCR-positive swabs using the previously described molecular methods, such as PCR-based serogrouping, PorA typing, FetA typing, and multilocus sequence typing (MLST) [9]. Antimicrobial susceptibility testing was performed using Etest strips (bioMérieux), and the minimum inhibitory concentration (MIC) was interpreted using the Clinical Laboratory Standards and Institute (CLSI) guideline [14]. The processing of specimens in this study is shown in Fig 1.

### Statistical analysis

This study was a descriptive study on *N*. *meningitidis* carriage. *N*. *meningitidis* carriers were defined as participants whose pharyngeal swabs showed an *N*. *meningitidis*-positive culture or real-time PCR. Continuous variables are presented as mean±standard deviation and categorical variables as numbers and percentages. Differences in proportion were tested with Fisher’s exact test. Factors associated with the carriage and acquisition of *N*. *meningitidis* were evaluated using univariate and multivariate analyses. Covariates of the multivariate logistic regression model included all significant risk factors in univariate analyses and other well-known risk factors. The results of univariate and multivariate analyses for identifying risk factors are reported as adjusted odds ratios (ORs) and 95% confidence intervals (CIs). Statistical tests were two-sided, and a *p*-value <0.05 was considered statistically significant. Data were analyzed using SPSS statistics 23 (IBM, Armonk, NY, USA).

## Results

### Carriage rates

**Fig 2:**
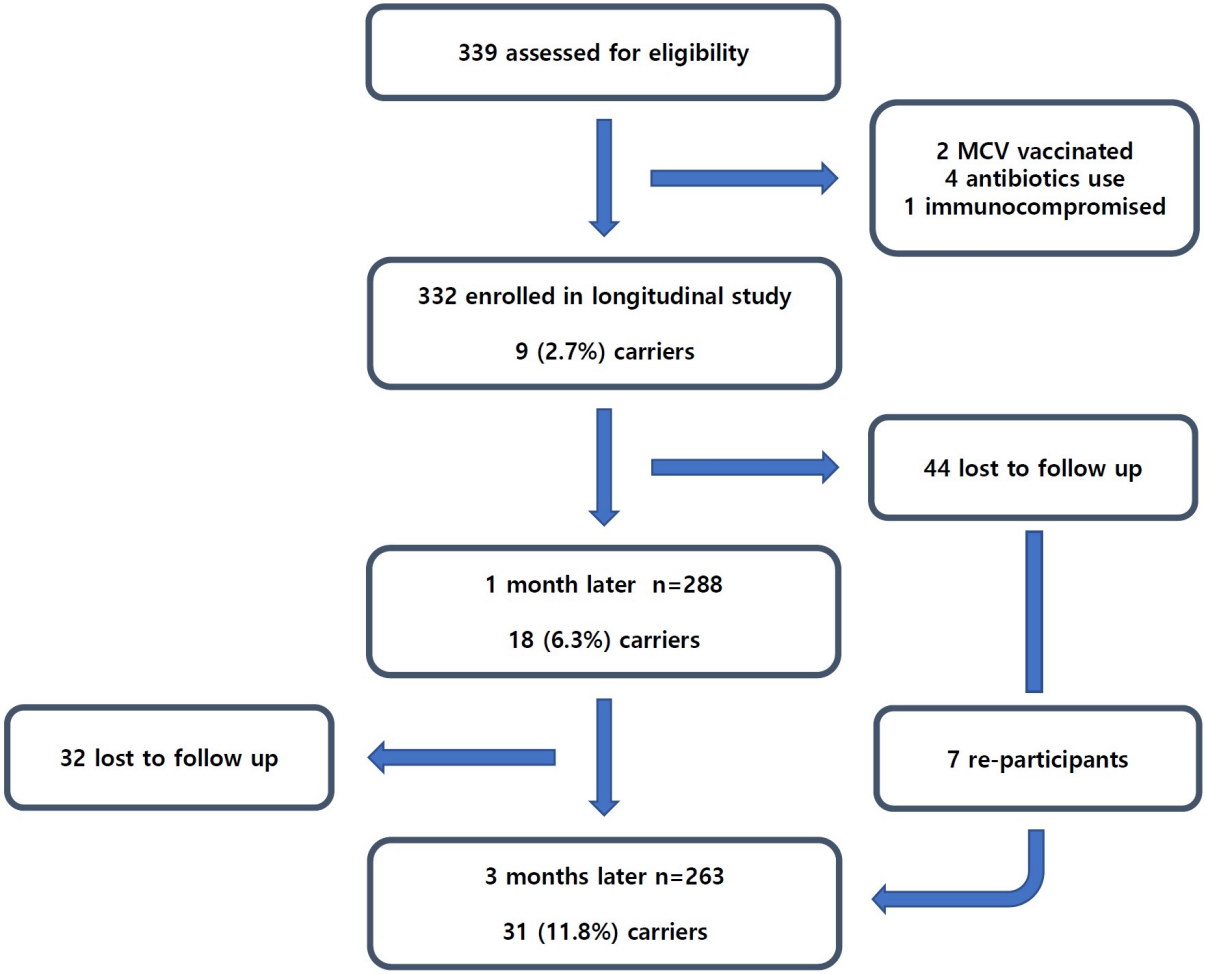
Flow chart of study participants.

**Fig 3:**
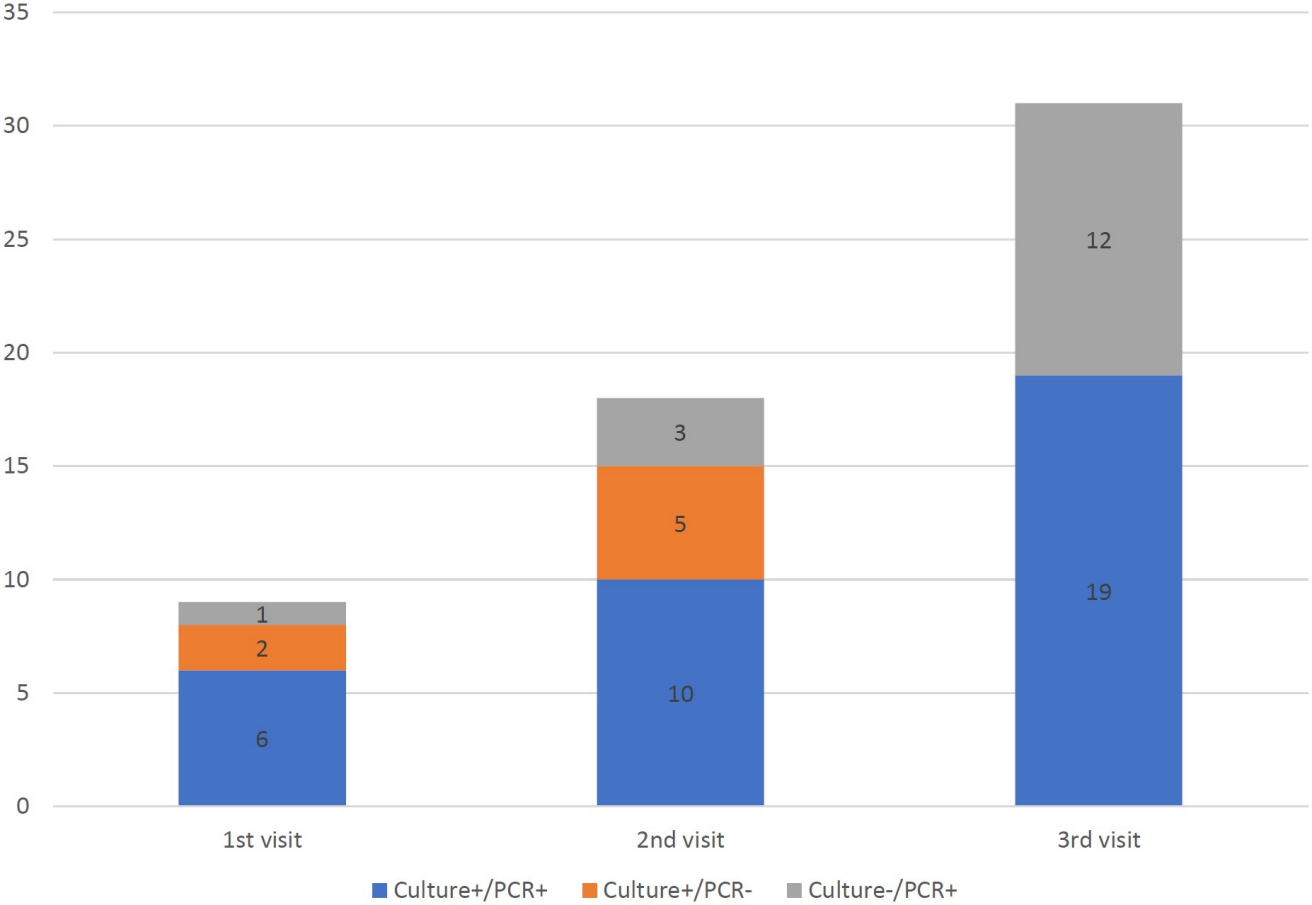
The number of *N*. *meningitidis*-positive results per visit as detected by each method.

We enrolled 332 first-year university students who provided samples at the first visit (Fig 2). The subjects’ median age at inclusion was 19 years (range 19–24), and 64.2% (213/332) were male. The dormitory consisted of two buildings, dormitory A and B, with 157 and 133 enrolled students, respectively. The proportion of students who participated in the second and third visits was 86.7% (288/332) and 79.2% (263/332), respectively. During the study period, 12.7% of participants (42/332, 95% CI 9.0–16.2%) were identified as carriers at least once. Meningococcal carriage rates increased from 2.7% (9/332, 95% CI 0.9–4.4%), to 6.3% (18/288, 95% CI 3.4–9.0%), and 11.8% (31/263, 95% CI 7.8–15.6%) at baseline, 1 month, and 3 months, respectively (Table 1, Fig 3). Of the carriers identified at baseline, 44.4% (4/9) still had positive results at the third visit with identical MLST. Of the 287 non-carriers who were followed up, 11.5% (33/287) became carriers while 88.5% (254/287) remained non-carriers. Two and five participants who had positive results on the first and second visits, respectively, had a negative conversion by the third visit. No case of IMD occurred during the follow-up period.

**Table 1:** Carriage rates of *Neisseria meningitidis* during follow-up period.

Timepoints	Swabbed (number of subjects)	Positive for *N*. *meningitides* by each method (number of subjects)			Carriage rates (percentage, 95% CI)
		Culture-positive/PCR-positive	Culture-positive/PCR-negative	Culture-negative/PCR-positive	
During admission to the dormitory (March 2018)	332	6	2	1	2.7 (0.9–4.4)
1 month later (April 2018)	288	10	5	3	6.3 (3.4–9.0)
3 months later (June 2018)	263	19	0	12	11.8 (7.8–15.6)

### Risk factor analysis

Risk factor analysis of participant characteristics on *N*. *meningitidis* carriage in the study population is shown in Table 2. Univariate analysis showed no significant association between *N*. *meningitidis* carriage and current smoking, alcohol consumption, three or more roommates, international travel, recent antibiotics intake, recent upper respiratory tract infection, cup/cigarette sharing, and intimate contact. However, in multivariate analysis, male sex (OR 2.61, 95% CI 1.15–5.96) and recent pub or club visits (OR 3.70, 95% CI 1.54–8.92) were associated with *N*. *meningitidis* carriage during the study period. Factors associated with the acquisition of *N*. *meningitidis* for the 287 noncarriers at baseline were also significant for male sex (OR 3.58, 95% CI 1.34–9.57) and recent pub or club visits (OR 3.33, 95% CI 1.39–7.94), but they were not significant for a dormitory building (*p* = 0.344).

**Table 2:** Factors associated with meningococcal carriage in the studied population.

	Carriers (N = 42)	Non-carriers (N = 290)	Univariate analysis		Multivariate analysis	
			Odds ratio (95% CI)	*P*-value	Odds ratio (95% CI)	*P*-value
Associated factors	N (%)	N (%)				
Sex, male	34 (81.0)	179 (61.7)	2.635 (1.177–5.899)	0.016	2.613 (1.145–5.961)	**0.022**
Current smoking	3 (7.1)	40 (13.8)	0.481 (0.142–1.630)	0.326		
Alcohol consumption	41 (97.6)	258 (89.0)	5.085 (0.676–38.237)	0.098	1.912 (0.232–15.733)	0.546
Three or more roommates	30 (71.4)	195 (67.5)	1.205 (0.591–2.459)	0.724		
International travel*	5 (11.9)	45 (15.5)	0.736 (0.274–1.973)	0.650		
Recent antibiotics exposure‡	5 (11.9)	27 (9.3)	1.316 (0.477–3.630)	0.781		
Recent upper respiratory infection†	36 (85.7)	224 (77.2)	1.768 (0.714–4.378)	0.237	1.856 (0.729–4.722)	0.194
Cup/cigarette sharing†	19 (45.2)	102 (35.2)	1.523 (0.792–2.927)	0.231		
Recent visit to pub or club‡	35 (83.3)	156 (53.8)	4.295 (1.847–9.985)	<0.001	3.701 (1.536–8.919)	**0.004**
Recent intimate contact*	13 (31.0)	64 (22.1)	1.583 (0.778–3.222)	0.240	1.312 (0.628–2.740)	0.470
Reside in dormitory A	26 (61.9)	183 (55.1)	1.377 (0.708–2.675)	0.344		
dormitory B	16 (38.1)	149 (44.9)				

*Within the past 4 weeks;

^†^Within the previous week;

^‡^Within the past 2 weeks.

### Serogroups of carrier strains and antimicrobial susceptibility

Table 3 summarizes the serogroup distribution and putative transmission of the 58 meningococcal isolates from the 42 carriers during the study period. Most isolates (29/58, 50%) were not serogroupable. Serogroup B was identified in 14/58 (24.1%) isolates, followed by serogroup H (8/58, 13.8%), and serogroup C (7/58, 12.1%). Based on serotype and MLST analysis, meningococci were transmitted from six carriers to other students. Among the initial carriers, one carrier transmitted a serogroup B strain to two other students, and another carrier transmitted a serogroup C strain to three others. Although there were no epidemiologic data other than MLST data, the transmission of B and C serotypes occurred within the same dormitory residents.

**Table 3:** Serogroup distribution of 58 isolates at each of the study visits and putative transmission events.

Serogroup	1st visit	2nd visit	3rd visit	Total, N (%)
B	3	3	8	14 (24.1)
	B1, B:P1.18,25:F1-5:ST-7612(cc41/44)		B1, B1-1	
		B2, B:P1.21–2,28:F5-64:ST-5542(cc5542)	B2-1	
		**B3, B:P1.19,15:F3-6:ST-3091(cc269)**	**B3, B3-1, B3-2**	
C	1	5	1	7 (12.1)
	**C1, C:P1.22,14–6:F1-7:ST-11278(cc32)**	**C1, C1-1, C1-2, C1-3**	**C1**	
H	2	3	3	8 (13.8)
		H1, H:P1.19–5,15:F5-5:ST-178(cc178)	H1, H1-1	
Nongroupable	3	7	19	29 (50.0)
	NG1, NG:P1.22–1,1–5:F3-3:ST-ND(cc11940)	NG1, NG1-1	NG1-1	
**Total**	9	18	31	58 (100.0)

The 58 isolates were obtained from the 42 participants identified as meningococcal carriers.

Letters represent each serotype, and number/hyphen denotes isolates of the same MLST.

The antimicrobial susceptibility of the cultured isolates is shown in Table 4. Of the 58 isolates, 42 were culture-positive, and the susceptibility test was performed on 41 available isolates, of which 26 had low penicillin susceptibility and seven were penicillin-resistant. Of the 26 isolates with low penicillin susceptibility, nine, five, and four were identified as serogroups B, C, and H, respectively, while eight were nongroupable.

**Table 4:** Antimicrobial susceptibility of 41 cultured isolates by Etest.

Antibiotics	MIC range (mg/L)	Number of isolates (%)		
		Susceptible	Intermediate	Resistant
Penicillin	0.023–0.38	15 (36.6)	19 (46.3)	7 (17.1)
Ampicillin	0.032–0.5	16 (39.0)	25 (61.0)	0
Ceftriaxone	0.002–0.003	41 (100)	0	0
Meropenem	0.008–0.064	41 (100)	0	0
Ciprofloxacin	0.003–0.008	41 (100)	0	0
Minocycline	0.047–0.5	41 (100)	0	0
Rifampin	0.004–0.32	41 (100)	0	0

MIC, minimal inhibitory concentration.

### Distribution of MLST sequence type by FetA and PorA typing

Of the 58 PCR- or culture-positive isolates, only 30 isolates were recovered from the specimens; MLST was performed on all 30 isolates. MLST of the 30 isolates showed that the most common sequence type (ST) was ST3091 (7/30; 23.3%), with all of these isolates belonging to clonal complex 269 and serogroup B (Table 5). Of the seven isolates with ST3091, five were F3-6 by FetA typing, while 19 and 15 subtypes by PorA typing. The second most common was clonal complex 178, consisting of ST178 and ST2397, all belonging to serogroup H, followed by clonal complex 32, consisting of ST11278 and one new sequence type. All clonal complex 32 isolates belonged to serogroup C and were not susceptible to penicillin and ampicillin. Interestingly, five isolates showed novel sequence types that were closely related to ST11940. All of the isolates in this clonal complex shared the same PorA1 types and were nongroupable.

**Table 5:** MLST, FetA typing, PorA typing, serogrouping and antimicrobial susceptibility of 30 isolates of *N*. *meningitides*.

Visit	Participant	MLST		FetA typing	PorA typing		Sero-group	MIC (mg/L) of						
		CC	ST		PorA1	PorA2		PEN	AMP	CRO	MEM	CIP	MIN	RFP
3		11	11	F1-1	5	2	NG	0.023	0.064	0.002	0.012	0.003	0.047	0.003
3			11	F1-1	5	2	NG	0.023	0.064	0.002	0.012	0.003	0.047	0.003
2	C1-2	32	11278	F1-7	22	14–6	C	0.38	0.38	0.002	0.032	0.004	0.19	0.032
2	C1-3		11278	F1-7	22	14–6	C	0.25	0.5	0.002	0.047	0.006	0.25	0.023
1, 2, 3	C1		11278	F1-7	22	14–6	C	0.25	0.38	0.002	0.047	0.006	0.5	0.32
2			NEW	F1-7	18–24	25	C	0.25	0.5	0.002	0.047	0.003	0.25	0.19
1, 3	B1	41/44	7612	F1-5	18	25	B	0.125	0.25	0.003	0.023	0.004	0.19	0.047
1		162	162	F5-9	7–2	4	B	0.064	0.047	0.002	0.012	0.003	0.125	0.032
2, 3	H1	178	178	F5-5	19–5	15	H	0.023	0.094	0.002	0.012	0.004	0.047	0.004
1, 2			178	F5-5	19	15	H	0.25	0.38	0.002	0.032	0.006	0.125	0.064
1			178	F5-5	19	15	H	0.25	0.25	0.002	0.032	0.006	0.19	0.094
3	H1-1		178	F5-5	19–5	15	H	0.032	0.125	0.002	0.016	0.006	0.064	0.003
3			178	Repeat	19–1	15	H	0.032	0.047	0.002	0.008	0.004	0.094	0.032
2			2397	F1-7	19–1	15–10	H	0.38	0.5	0.002	0.047	0.006	0.125	0.032
2, 3		198	2033	F3-73 (78.2)	18–17 (32.7)	25–6	NG	0.38	0.38	0.002	0.032	0.003	0.19	0.047
3			2033	F3-73 (78.3)	18	25–6	NG	0.125	0.38	0.002	0.008	0.004	0.125	0.047
1		269	3091	F1-19	12–6	13–13	B	0.25	0.38	0.002	0.032	0.006	0.125	0.008
3			3091	F3-6	19–8	15–35	B	0.38	0.75	0.003	0.064	0.004	0.25	0.023
3	B3-1		3091	F3-6	19	15	B	0.064	0.064	0.002	0.012	0.004	0.125	0.008
3			3091	F3-6	19–8	15–35	B	0.38	0.75	0.002	0.064	0.004	0.125	0.008
3	B3-2		3091	F3-6	19	15	B	0.25	0.25	0.002	0.032	0.006	0.19	0.094
2, 3	B3		3091	F3-6	19	15	B	0.032	0.047	0.002	0.008	0.004	0.125	0.012
2			3091	F5-5	19	15	B	0.094	0.063	0.003	0.012	0.004	0.094	0.012
2	B2	5542	5542	F5-64	21–2	28	B	0.19	0.25	0.002	0.047	0.003	0.1	0.047
3	B2-1		5542	F5-64	21–2	28	B	0.25	0.5	0.002	0.032	0.004	1.0	0.032
1, 2, 3		11940	NEW	F3-3	22–1	2–48	NG	0.064	0.032	0.002	0.012	0.006	0.094	0.016
3			NEW	F3-3	22–1	1–5	NG	0.38	0.75	0.002	0.064	0.004	0.25	0.064
2, 3	NG1-1		NEW	F3-3	22–1	1–5	NG	0.032	0.064	0.002	0.008	0.004	0.19	0.094
1, 2	NG1		NEW	F3-3	22–1	1–5	NG	0.25	0.5	0.002	0.064	0.008	0.19	0.094
3			NEW	F1-19	22–1	2–48	NG	0.19	0.38	0.003	0.032	0.006	0.25	0.125

In the case of multiple visits, the same isolate was identified in one participant.

Participants were classified according to the putative transmission events in Table 3, and the specimens of B1-1 and C1-1 could not be cultured.

Antimicrobial susceptibility: white cell, susceptible; gray cell, intermediate; black cell, resistant.

AMP, ampicillin; CC, clonal complex; CIP, ciprofloxacin; CRO, ceftriaxone; MEM, meropenem; MIC, minimal inhibitory concentration; MIN, minocycline; MLST, multilocus sequence typing; NG, nongroupable; PEN, penicillin; RFP; rifampin; ST, sequence type.

## Discussions

South Korea’s national notifiable diseases surveillance system has reported a relatively low incidence of meningococcal disease, 0.01–0.08 cases per 100,000 persons. Nonetheless, previous reports on the seroprevalence and carriage rate of *N*. *meningitidis* in South Korean populations suggested a non-negligible burden of meningococcal disease in the country [1, 9, 15–17]. *N*. *meningitidis* is a leading cause of bacterial meningitis in adolescents and young adults. Moreover, university students, especially those living in dormitories, are known to have an increased risk of meningococcal disease. This longitudinal study showed that *N*. *meningitidis* carriage rates significantly increased from 2.7% to 11.8% over the first semester among new university entrants in South Korea who were living in dormitories. In the isolates swabbed at the third visit, there were culture-negative, PCR-positive samples (n = 13), all of which were *sodC*-positive and *ctrA*-negative. The genotypes were not identified at the first and second visits, and they were not included in the analysis as they were likely to be false-positive. However, the possibility of actual expansion of NG meningococci could not be completely ruled out, and the carriage rate increased to 16.7% (44/263) when the third visit included culture-negative, *sodC*-positive swabs.

Previous studies of *N*. *meningitidis* carriage in South Korea have shown a carriage rate of 3.4% in healthy Korean adolescents, with serogroup B being the most common serogroup, followed by serogroups C, E, and Y [15]. In contrast, another study conducted on first-year university students in South Korea in 2009 did not show a significant increase in carriage rates in a different university dormitory [16]. Such contrasting observations might have resulted from the longer time interval between visits and the larger sample size in our study. More studies should be performed to clarify the transmission dynamics of *N*. *meningitidis* among university students in South Korea.

In the present study, nongroupable isolates were the most prevalent, followed by serogroup B. Similarly, Lee *et al*. evaluated the serogroup changes of *N*. *meningitidis* in South Korea between 2010 and 2016, and showed that the most common serogroup was serogroup B, which was isolated every year except for 2011. Moreover, Kim *et al*. found that serogroup B was the most common serogroup in Korean adolescents [9, 15]. Additionally, Serogroup B has been shown to be the predominant serotype in carriage studies conducted in Europe and the United States (U.S.) [18–20]. Conversely, the most recent meningococcal disease outbreak was reported in a South Korean army basic training center in 2011, and this case was of serogroup W. Thereafter, an MCV4 vaccination program was implemented for all new military recruits in South Korea [21].

Despite the previous studies on seroprevalence and carriage rates of *N*. *meningitidis* in the South Korean population, the necessity of vaccinating specific populations such as college students is not well-defined. A study conducted in the U.S. showed that the overall incidence of meningococcal disease among university students was comparable to that of the general population; however, the disease rates among students living in dormitories were higher compared to those living off campus. Consequently, the Advisory Committee on Immunization Practices and the Committee on Infectious Diseases of the American Academy of Pediatrics recommended meningococcal conjugate vaccination for incoming college freshmen [22, 23]. Although meningococcal vaccines are not included in the national immunization program for children in South Korea, MCV4 is currently available and recommended by the KCDC for immunocompromised individuals, travelers to endemic areas, laboratory workers, new military recruits, and university freshmen who plan to live in a dormitory [10, 21]. Since South Korea has a conscription system, male adults must enlist in the military and receive MCV4. About 300,000 new recruits are estimated to be vaccinated annually; however, since male freshmen tend to enter military service after attending school for more than a year, herd immunity is not formed in the dormitory. While asymptomatic carriage rates do not necessarily indicate the potential for invasive disease, our study suggests that it is necessary to consider meningococcal vaccination for South Korean students before dormitory admission, given the importance of maintaining herd immunity.

The cultured isolates in this study were susceptible to most antibiotics, including ceftriaxone, ciprofloxacin, and rifampin, which are used as prophylactic antibiotics for contacts. However, 63.4% of the isolates showed low susceptibility to penicillin, which is recommended as a first-line drug in IMD. These results were similar to those of a previous study conducted in South Korea, in which 78% of the isolates showed low penicillin-susceptibility [9]. In the aforementioned study, penicillin resistance was reported in serogroups W and C. Although serogroup W was not isolated in our study, resistance was reported in all serogroups, including B; therefore, the spread of resistance should be a concern.

Our study has some limitations. First, the results may not represent the epidemiology of meningococcal carriage among university students in South Korea, since this study was performed on a small number of subjects within a university campus. There may also be some selection bias, as only students who signed informed consent were enrolled in the study. Moreover, seasonal variation could not be evaluated, since the study was performed between March and June. In addition, recall bias may affect the results, as we collected data based on the subjects’ responses to the questionnaire. Although the same genotype propagation was determined based on MLST, this study was limited as whole-genome sequencing was not implemented and other epidemiologic grounds were not provided.

In conclusion, *N*. *meningitidis* carriage rates among new university entrants who lived in a dormitory in South Korea significantly increased during the first semester, probably due to the transmission of an identical genotype among students. Based on the risk of meningococcal disease, meningococcal vaccination should be considered for students before dormitory admission.

## Supporting information

S1 File This is the questionnaire for study participants in Korean. (DOCX)

S2 File This is the questionnaire for study participants in English. (DOCX)
