# Supplementary material for: Longitudinal study of meningococcal carriage rates in university entrants living in a dormitory in South Korea
Source: PLoS One. 2021 Jan 28;16(1):e0244716. doi: 10.1371/journal.pone.0244716 (PMC7842983; doi:10.1371/journal.pone.0244716)
Supplement: S1 File — (DOCX) [file pone.0244716.s001.docx]

설문지

국내 1개 대학교 신입생에서 기숙사 입사 전후

수막구균 보균율 변화 연구

| 피험자 번호 | 성명 (이니셜) |
| --- | --- |
|  |  |

우선, 본 연구에 관심을 가져 주셔서 감사드립니다.

본 설문지를 통하여 제공되는 귀하의 개인 신상 정보는 공개되지 않으며, 연구 기록의 열람은 본 연구 과제에 국한되며 연구 목적으로만 사용될 것입니다.

이 페이지에서 드리는 질문은 귀하께서 연구 대상자로서 적합한지를 묻는 질문입니다.

만약 하나라도 “아니오”에 해당하시는 항목이 있으시다면 본 연구에 참여하실 수 없습니다. 이 페이지의 모든 질문 항목에 “예”라고 표기하신 경우에만 다음장으로 진행하여 주시기 바랍니다.

|  | **예** | **아니오** |
| --- | --- | --- |
| 01) 귀하께서는 만 연세대학교 1학년으로서 1학기 이상 기숙사 생활을 할 예정이신가요? |  |  |
| 02) 귀하께서 만 19세 이상일 경우에는 본인이, 만 19세 미만일 경우에는 보호자 또는 법정 대리인이 본 연구와 관련된 모든 설명을 듣고 이해하였으며, 본 연구에 참여하기를 자발적인 의사에 의하여 동의하고 동의서를 작성하셨나요? |  |  |

다음 질문은 귀하의 기본 정보와 관련된 질문입니다. 병역 관련 질문은 남자분만 답하시기 바랍니다.

| **성별** | **생년월일** | |
| --- | --- | --- |
| 남 □  여 □ | 년 월 일 | |
|  | **나이** | 만 세 |
| **병역** | 미필 또는 면제 □  군필 □ (복무기간: 년 월 ~ 년 월) | |

다음 질문은 귀하의 예방 접종력과 관련된 질문입니다. 접종일이 정확하게 기억이 나지 않으신다면 기억나시는 부분까지 작성하여 주시기 바랍니다.

|  | **예** | **아니오** |
| --- | --- | --- |
| 01) 귀하께서는 수막구균 예방 접종을 받으신 적이 있으신가요? |  |  |
| “**예**”라고 표기하신 분만 답하세요.   - 1. 접종일 년 월 일 | | |
| 02) 귀하께서는 최근 5년 이내에 기타 예방 접종을 받으신 적이 있으신가요? |  |  |
| “**예**”라고 표기하신 분만 답하세요.   - 1. 접종명   2. 접종일 년 월 일 | | |

다음 질문은 귀하의 과거 병력과 관련된 질문입니다. 정보가 정확하게 기억이 나지 않으신다면 기억나시는 부분까지 작성하여 주시기 바랍니다.

|  | **예** | **아니오** |
| --- | --- | --- |
| 01) 귀하께서는 과거에 특정 질환을 진단받으신 적이 있으신가요? |  |  |
| “**예**”라고 표기하신 분만 답하세요.   - 1. 병명은 무엇인가요?   2. 진단일: 년 월 일   3. 현재 상태는 어떠한가요?   정기적 병원 방문 또는 약물복용 □ 완치 □ | | |
| 02) 귀하께서는 과거에 수술 또는 시술을 받으신 적이 있으신가요? |  |  |
| “**예**”라고 표기하신 분만 답하세요.   - 1. 수술명 또는 시술은 무엇인가요?   2. 시행일: 년 월 일 | | |

다음 질문은 기숙사에서 귀하의 생활 방식과 관련된 질문입니다. 정보가 정확하게 기억이 나지 않으신다면 기억나시는 부분까지 작성하여 주시기 바랍니다.

|  | **Yes** | **No** |
| --- | --- | --- |
| 01) 연세대학교 기숙사생이신가요? |  |  |
| “**예**”라고 표기하신 분만 답하세요.  03-01) 귀하의 방의 위치는 어디인가요? 학사 층  03-02) 귀하는 귀하를 포함하여 몇 명의 룸메이트와 생활하시나요? 명  03-03) 귀하가 기숙사 생활을 하신지는 얼마나 되셨나요? 개월 | | |

다음 질문은 귀하의 약물 복용력과 관련된 질문입니다. 정보가 정확하게 기억이 나지 않으신다면 기억나시는 부분까지 작성하여 주시기 바랍니다.

|  | **예** | **아니오** |
| --- | --- | --- |
| 01) 귀하께서는 최근 2주 이내에 경구 또는 주사 항생제를 복용하신 적이 있으신가요? |  |  |
| “**예**”라고 표기하신 분만 답하세요.   - 1. 약이름 및 용량   2. 복용 사유:   3. 복용 기간: 년 월 일 ~ 년 월 일 | | |
| 02) 귀하께서는 최근 2주 이내에 경구 또는 주사 스테로이드 제재를 투여받으신 적이 있으신가요?  (스테로이드 연고, 안약등은 제외) |  |  |
| “**예**”라고 표기하신 분만 답하세요.   - 1. 약이름 및 용량   2. 복용 사유:   3. 복용 기간: 년 월 일 ~ 년 월 일 | | |
| 03) 귀하께서 최근 2주 이내에 투여받으신 기타 약물이 있으신가요? |  |  |
| “**예**”라고 표기하신 분만 답하세요.   - 1. 약이름 및 용량   2. 복용 사유:   3. 복용 기간: 년 월 일 ~ 년 월 일 | | |

다음 질문은 귀하의 현재 상태와 관련된 질문입니다. 정보가 정확하게 기억이 나지 않으신다면 기억나시는 부분까지 작성하여 주시기 바랍니다.

|  | | **예** | **아니오** |
| --- | --- | --- | --- |
| 01) 귀하는 현재 흡연자이신가요? | |  |  |
| “**예**”라고 표기하신 분만 답하세요.   - 1. 하루 흡연량: 갑   2. 흡연 기간: 년 | “**아니오**”라고 표기하신 분만 답하세요.   - 1. 한번도 피워본 적이 없다 □   2. 년 전에 끊었다. □ | | |
| 02) 귀하는 음주를 하시나요? | |  |  |
| “**예**”라고 표기하신 분만 답하세요  02-01) 1회 음주량: 병  02-02) 1주일에 음주 횟수: 회/주 | | | |
| 03) 귀하께서는 최근 1주 이내에 감기 증상을 느끼신 적이 있으신가요? | |  |  |
| “**예**”라고 표기하신 분만 답하세요.   - 1. 해당 증상에 모두 표기 바랍니다.   기침 □ 가래 □ 콧물 □ 코막힘 □ 재채기 □  인후통 □ 이물감 □ 건조감 □ 발열 □ 전신통 □  기타:   - 1. 증상 지속 기간: : 년 월 일 ~ 년 월 일   2. 상기 증상에 대한 조치는 무엇이었나요?   경과 관찰 □ 약물 치료 (약국) □ 약물 치료 (병원) □ | | | |

다음 질문은 귀하의 생활 습관과 관련된 질문입니다. 정보가 정확하게 기억이 나지 않으신다면 기억나시는 부분까지 작성하여 주시기 바랍니다.

|  | **예** | **아니오** |
| --- | --- | --- |
| 01) 귀하께서는 최근 4주 이내에 외국을 다녀오신 적이 있으신가요? |  |  |
| “**예**”라고 표기하신 분만 답하세요.   - 1. 출국일: 년 월 일   2. 체류 했던 국가/도시/체류기간  1. / / 년 월 일 ~ 년 월 일 2. / / 년 월 일 ~ 년 월 일 3. / / 년 월 일 ~ 년 월 일 4. / / 년 월 일 ~ 년 월 일 5. / / 년 월 일 ~ 년 월 일    1. 입국일: 년 월 일 | | |
| 02) 귀하께서는 최근 2주 이내에 술집 또는 클럽을 방문하신 적이 있으신가요? |  |  |
| - 1. 평상 시 1주일에 술집 또는 클럽 방문 횟수: 회/주   “**예**”라고 표기하신 분만 답하세요.   - 1. 최근 2주간 술집 또는 클럽 방문 횟수: 회 | | |

|  | **예** | **아니오** |
| --- | --- | --- |
| 03) 귀하께서 최근 1주 이내에 물컵, 술잔, 수저, 담배등을 타인과 공유하신 적이 있으신가요? |  |  |
| - 1. 평상 시 1주일간 공유 빈도   3회 미만 □ 3~6회 □ 7~9회 □ 10회 이상 □  “**예**”라고 표기하신 분만 답하세요.   - 1. 최근 1주 이내에 공유 빈도   3회 미만 □ 3~6회 □ 7~9회 □ 10회 이상 □ | | |
| 04) 귀하께서는 최근 4주 이내에 키스 등의 접촉을 한 적이 있으신가요? |  |  |
| “**예**”라고 표기하신 분만 답하세요.   - 1. 최근 4주간 키스 등의 접촉 횟수: 회 | | |

수고 많으셨습니다.

귀하의 개인 정보는 보호될 것이며, 귀하가 작성하여 주신 정보는 추후 국내 수막구균 예방접종 지침을 마련하는 데에 있어 중요한 자료로서 기여될 것입니다.

적지 않은 수의 질문에 성심 성의껏 답해 주시고 본 연구에 참여해 주신 점 감사드립니다.
