# Supplementary material for: Longitudinal study of meningococcal carriage rates in university entrants living in a dormitory in South Korea
Source: PLoS One. 2021 Jan 28;16(1):e0244716. doi: 10.1371/journal.pone.0244716 (PMC7842983; doi:10.1371/journal.pone.0244716)
Supplement: S2 File — (DOCX) [file pone.0244716.s002.docx]

Questionnaire for

Longitudinal study of meningococcal carriage rates in university entrants living in a dormitory in South Korea

| No. | Name (initials) |
| --- | --- |
|  |  |

First of all, thank you for your interest in this study. Your personally identifiable information provided through this questionnaire will not be disclosed, and access to research records is limited to this research project and will be used for research purposes only.

The questions on this page are asking if you are suitable as a study subject. If any of the items are “No”, you cannot participate in this study. Please proceed to the next page only if you have indicated “yes” to all of the question items on this page.

|  | **Y** | **N** |
| --- | --- | --- |
| 01) Are you going to live in a dormitory for more than one semester as a freshman at Yonsei University? |  |  |
| 02) If you are 19 years of age or older, you have heard and understood all explanations related to this study, and if you are under the age of 19, did you voluntarily agree to participate in this study and write consent? |  |  |

The following questions are related to your basic information. Only men should answer questions related to military service.

| **Sex** | **Birth date** | |
| --- | --- | --- |
| Men □  Women □ | year month day | |
|  | **age** |  |
| **Military service** | Not completed or exempted □  Completed □ (duration: y m ~ y m) | |

The following questions are related to your vaccination history. If you do not remember the vaccination date correctly, please fill out the part you remember.

|  | **Y** | **N** |
| --- | --- | --- |
| 01) Have you ever been vaccinated against meningococci? |  |  |
| Answer only those who marked “yes”.   - 1. Date y m d | | |
| 02) Have you had any other vaccinations in the last 5 years? |  |  |
| Answer only those who marked “yes”.   - 1. Vaccination   2. Date y m d | | |

The following questions are related to your past medical history. If you do not remember the information correctly, please fill out the part you remember.

|  | **Y** | **N** |
| --- | --- | --- |
| 01) Have you ever been diagnosed with a specific condition in the past? |  |  |
| Answer only those who marked “yes”.   - 1. What is the disease name?   2. Diagnosis date: y m d   3. What is your current status?   Regular hospital visits or medication □ Cured □ | | |
| 02) Have you ever had an operation or procedure in the past? |  |  |
| Answer only those who marked “yes”   - 1. What is the name of the operation or procedure?   2. Date: y m d | | |

The following questions are related to your lifestyle in dorms. If you do not remember the information correctly, please fill out the part you remember.

|  | **Y** | **N** |
| --- | --- | --- |
| 01) Are you a dormitory student at Yonsei University? |  |  |
| Answer only those who marked “yes”.  03-01) Where is your room located? building floor  03-02) How many roommates do you live with, including you?  03-03) How long have you been in dorms? m | | |

The following questions are related to your medication history. If you do not remember the information correctly, please fill out the part you remember.

|  | **Y** | **N** |
| --- | --- | --- |
| 01) Have you ever taken oral or injectable antibiotics within the last 2 weeks? |  |  |
| Answer only those who marked “yes”.   - 1. Medication name, dosage   2. Reason for taking:   3. Duration: y m d ~ y m d | | |
| 02) Have you ever been given an oral or injectable steroid formulation within the last 2 weeks? (Steroid ointment, eye drops are excluded) |  |  |
| Answer only those who marked “yes”.   - 1. Medication name, dosage   2. Reason for taking:   3. Duration: y m d ~ y m d | | |
| 03) Do you have any other medications you have been given within the last 2 weeks? |  |  |
| Answer only those who marked “yes”.   - 1. Medication name, dosage   2. Reason for taking:   3. Duration: y m d ~ y m d | | |

The following questions are related to your current status. If you do not remember the information correctly, please fill out the part you remember.

|  | | **Y** | **N** |
| --- | --- | --- | --- |
| 01) Are you currently a smoker? | |  |  |
| Answer only those who marked “yes”.   - 1. Amount per day: pack   2. Duration: y | Answer only those who marked “no”.   - 1. Never smoked □   2. Quit y ago □ | | |
| 02) Do you drink alcohol? | |  |  |
| Answer only those who marked “yes”.  02-01) Drinking amount per time: bottle  02-02) Number of times you drink alcohol per week: times/week | | | |
| 03) Have you ever had any cold symptoms within the last week? | |  |  |
| Answer only those who marked “yes”.   - 1. Please mark all of the symptoms.   cough □ sputum □ rhinorrhea □ stuffed nose □ sneeze □  sore throat □ foreign body sensation □ feeling dry □ fever □ myalgia □  etc.:   - 1. duration: y m d ~ y m d   2. What was the measure for the above symptoms??   observation □ medication (pharmacy) □ medication (clinic) □ | | | |

The following questions are related to your lifestyle. If you do not remember the information correctly, please fill out the part you remember.

|  | **Y** | **N** |
| --- | --- | --- |
| 01) Have you been abroad in the last 4 weeks? |  |  |
| Answer only those who marked “yes”.   - 1. Departure date: y m d   2. Country/City/Duration of stay  1. / / y m d ~ y m d 2. / / y m d ~ y m d 3. / / y m d ~ y m d 4. / / y m d ~ y m d 5. / / y m d ~ y m d    1. Arrival date: y m d | | |
| 02) Have you ever visited a pub or club within the last 2 weeks? |  |  |
| - 1. Number of visits to a pub or club usually per week: times/week   Answer only those who marked “yes”.   - 1. Number of visits to a pub or club last 2 weeks: times | | |

|  | **Y** | **N** |
| --- | --- | --- |
| 03) Have you shared water cups, drinking glasses, spoons, cigarettes, etc. with others within the last week? |  |  |
| - 1. Sharing frequency usually per week   Less than 3 times □ 3~6 times □ 7~9 times □ More than 10 times □  Answer only those who marked “yes”.   - 1. Sharing frequency within the last week   Less than 3 times □ 3~6 times □ 7~9 times □ More than 10 times □ | | |
| 04) Have you ever had any intimate contact within the last 4 weeks? |  |  |
| Answer only those who marked “yes”.   - 1. Number of intimate contacts in the last 4 weeks: 회 | | |

You did a really good job.

Your personal information will be protected, and the information you provide will serve as an important resource in preparing guidelines for domestic meningococcal vaccination in the future.

Thank you for your sincerely answering the not few questions and for your participation in this study.
